# 1-{3-[1-(Hydroxyimino)ethyl]-4-methyl-1*H*-pyrazol-5-yl}ethanone

**DOI:** 10.1107/S1600536811036518

**Published:** 2011-09-14

**Authors:** Sergey Malinkin, Larysa Penkova, Vadim A. Pavlenko, Matti Haukka, Svetlana V. Pavlova

**Affiliations:** a Kiev National Taras Shevchenko University, Department of Chemistry, Volodymyrska Str. 64, 01601 Kiev, Ukraine; b University of Joensuu, Department of Chemistry, PO Box 111, FI-80101 Joensuu, Finland

## Abstract

In the title compound, C_8_H_11_N_3_O_2_, the oxime and the acetyl groups adopt a transoid conformation, while the pyrazole H atom is localized in the proximity of the acetyl group and is *cis* with respect to the acetyl O atom. In the crystal, dimers are formed as the result of hydrogen-bonding inter­actions involving the pyrazole NH group of one mol­ecule and the carbonyl O atom of another. The dimers are associated into sheets *via* O—H⋯N hydrogen bonds involving the oxime hydroxyl and the unprotonated pyrazole N atom, generating a macrocyclic motif with six mol­ecules.

## Related literature

For details and applications of related pyrazoles, see: Kovbasyuk *et al.* (2004 ▶); Krämer & Fritsky (2000 ▶); Sachse *et al.* (2008 ▶). For the use of azomethine-functionalized pyrazoles in coordination chemistry and catalysis, see: De Geest *et al.* (2007 ▶); Roy *et al.* (2008 ▶). For the use of the oxime substituents in the synthesis of polynucleative ligands, see: Kanderal *et al.* (2005 ▶); Moroz *et al.* (2010 ▶). For related structures, see: Fritsky *et al.* (1998 ▶); Mokhir *et al.* (2002 ▶); Petrusenko *et al.* (1997 ▶); Sliva *et al.* (1997 ▶); Świątek-Kozłowska *et al.* (2000 ▶); Wörl *et al.* (2005*a* ▶,*b* ▶). For the preparation of related ligands, see: Wolff (1902 ▶).
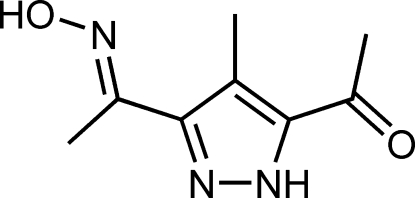


## Experimental

### 

#### Crystal data

C_8_H_11_N_3_O_2_
*M* *_r_* = 181.20
Monoclinic, 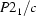

*a* = 9.0721 (2) Å
*b* = 11.7030 (7) Å
*c* = 8.2401 (9) Å
β = 104.124 (3)°
*V* = 848.41 (11) Å^3^
*Z* = 4
Mo *K*α radiation
μ = 0.11 mm^−1^
*T* = 120 K
0.46 × 0.33 × 0.13 mm

#### Data collection

Nonius KappaCCD diffractometer
Absorption correction: multi-scan (*DENZO*/*SCALEPACK*; Otwinowski & Minor, 1997 ▶) *T*_min_ = 0.955, *T*_max_ = 0.987
8532 measured reflections
1925 independent reflections
1486 reflections with *I* > 2σ(*I*)
*R*_int_ = 0.061

#### Refinement

*R*[*F* ^2^ > 2σ(*F* ^2^)] = 0.115
*wR*(*F* ^2^) = 0.346
*S* = 1.12
1925 reflections
125 parameters
H-atom parameters constrained
Δρ_max_ = 0.57 e Å^−3^
Δρ_min_ = −0.50 e Å^−3^

### 

Data collection: *COLLECT* (Nonius, 1998 ▶); cell refinement: *DENZO*/*SCALEPACK* (Otwinowski & Minor, 1997 ▶); data reduction: *DENZO*/*SCALEPACK*; program(s) used to solve structure: *SIR2004* (Burla *et al.*, 2005 ▶); program(s) used to refine structure: *SHELXL97* (Sheldrick, 2008 ▶); molecular graphics: *ORTEP-3* (Farrugia, 1997 ▶); software used to prepare material for publication: *SHELXL97*.

## Supplementary Material

Crystal structure: contains datablock(s) I, global. DOI: 10.1107/S1600536811036518/hy2464sup1.cif

Structure factors: contains datablock(s) I. DOI: 10.1107/S1600536811036518/hy2464Isup2.hkl

Supplementary material file. DOI: 10.1107/S1600536811036518/hy2464Isup3.cml

Additional supplementary materials:  crystallographic information; 3D view; checkCIF report

## Figures and Tables

**Table 1:** Hydrogen-bond geometry (Å, °)

*D*—H⋯*A*	*D*—H	H⋯*A*	*D*⋯*A*	*D*—H⋯*A*
O1—H1*O*⋯N2^i^	1.05	1.89	2.932 (6)	170
N3—H3*N*⋯O2^ii^	0.88	2.00	2.840 (5)	157

Symmetry codes: (i) 
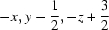
; (ii) 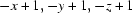.

**Fig. 1.**
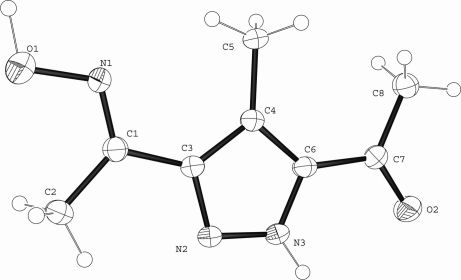
The molecular structure of the title compound, with displacement ellipsoids drawn at the 40% probability level.

**Fig. 2.**
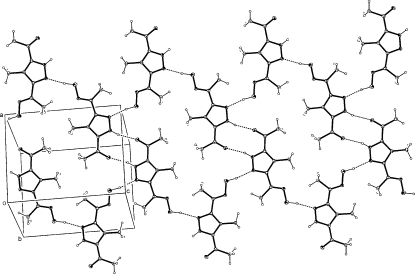
A portion of the crystal packing. Intermolecular hydrogen bonds (dashed lines) link the molecules into a two-dimensional network.

## supplementary crystallographic information

### Comment

Pyrazole-based ligands have attracted considerable attention due to their bridging nature and possibility for easy functionalization with various additional donor groups (Kovbasyuk *et al.*, 2004; Krämer & Fritsky, 2000; Sachse *et al.*, 2008). In particular, azomethine-functionalized pyrazoles have been used extensively as ligands in the field of coordination chemistry and catalysis (De Geest *et al.*, 2007; Roy *et al.*, 2008). Furthermore, introduction of the potentially bridging oxime group into the ligands already having bridging moieties (such as pyrazoles) may result in significant increase of coordination versatility of such ligands and afford the formation of metal complexes of high nuclearity and coordination polymers (Kanderal *et al.*, 2005; Moroz *et al.*, 2010). The title compound, having different substituents in the 3- and 5-positions of the pyrazole ring (the oxime and the acetyl groups) was synthesized as a part of our study of the abovementioned ligands and we report herein its crystal structure.

In the title compound (Fig. 1), the oxime and acetyl groups are in the transoid conformation in reference to one another, while the pyrazole proton is localized in the proximity of the acetyl group and is cis with respect to the acetyl O atom. The molecule is virtually planar, with the maximal deviation from the mean plane defined by the non-hydrogen atoms not exceeding 0.047 (5) Å for the methyl C5. The C—C, C—N and N—N bond lengths in the pyrazole ring are normal for the 3,5-disubstituted pyrazoles (Petrusenko *et al.*, 1997, Wörl *et al.*, 2005*a*,*b*). The bond lengths and angles within the acetyl and oxime groups are normal and comparable to those in the related structures (Fritsky *et al.*, 1998; Mokhir *et al.*, 2002; Świątek-Kozłowska *et al.*, 2000). The C, N, O atoms of the oxime group exist in the nitroso-form (Mokhir *et al.*, 2002; Sliva *et al.*, 1997).

The crystal of the title compound has a layer structure formed entirely by hydrogen bonds between the molecules. The approximately planar dimers form as the result of hydrogen-bonding interactions (Table 1) involving the pyrazole NH group of one molecule and the carbonyl O atom of another. The dimers are associated into planar sheets via O—H···N hydrogen bonds involving the unprotonated pyrazole N atom and the oxime hydroxyl, generating a macrocyclic motif with six molecules (Fig. 2).

### Experimental

3,5-Di-acetyl-4-methyl-1*H*-pyrazole (Wolff, 1902) (0,30 g, 1.81 mmol), NH_2_OH.HCl (0.09 g, 1.3 mmol) and sodium acetate (0.14 g, 1.3 mmol) were dissolved in water (10 ml). The mixture was stirred for 2 h, and the pH value was adjusted to 4 by slow addition of aqueous HCl (1:1). The formed precipitate was separated by filtration and purified by recrystallization from water/methanol (v/v, 1:1). Yield: 0.10 g (30 %). Analysis, calculated for C_8_H_11_N_3_O_2_: C 53.03, H 6.12, N 23.19%; found: C 52.72, H 6.32, N 23.25%. The water solution of the title compound was allowed to evaporate slowly over several days. Yellow crystals suitable for single-crystal X-ray diffraction were collected.

### Refinement

The crystal structure was refined with two twin components (twin matrices: 1 0 0.537 0 -1 0 0 0 -1 and 1.008 0 0.502 0 -1 0 -0.033 0 -1.008). BASF values were refined to 0.241 and 0.069, respectively. H atoms bonded to N and O atoms were located from a difference Fourier map but constrained to ride on their parent atoms, with *U*_iso_(H) = 1.2*U*_eq_(N) and 1.5*U*_eq_(O). H atoms of the methyl groups were positioned geometrically and refined as riding atoms, with C—H = 0.98 Å and with *U*_iso_(H) = 1.5*U*_eq_(C).

### Crystal data

**Table:**

C_8_H_11_N_3_O_2_	*F*(000) = 384
*M**_r_* = 181.20	*D*_x_ = 1.419 Mg m^−^^3^
Monoclinic, *P*2_1_/*c*	Mo *K*α radiation, λ = 0.71073 Å
Hall symbol: -P 2ybc	Cell parameters from 1467 reflections
*a* = 9.0721 (2) Å	θ = 3.0–27.5°
*b* = 11.7030 (7) Å	µ = 0.11 mm^−^^1^
*c* = 8.2401 (9) Å	*T* = 120 K
β = 104.124 (3)°	Block, yellow
*V* = 848.41 (11) Å^3^	0.46 × 0.33 × 0.13 mm
*Z* = 4	

### Data collection

**Table:**

Nonius KappaCCD diffractometer	1925 independent reflections
Radiation source: fine-focus sealed tube	1486 reflections with *I* > 2σ(*I*)
horizontally mounted graphite crystal	*R*_int_ = 0.061
Detector resolution: 9 pixels mm^-1^	θ_max_ = 27.5°, θ_min_ = 2.9°
φ and ω scans with κ offset	*h* = −11→11
Absorption correction: multi-scan (*DENZO*/*SCALEPACK*; Otwinowski & Minor, 1997)	*k* = −14→15
*T*_min_ = 0.955, *T*_max_ = 0.987	*l* = −10→10
8532 measured reflections	

### Refinement

**Table:**

Refinement on *F*^2^	Primary atom site location: structure-invariant direct methods
Least-squares matrix: full	Secondary atom site location: difference Fourier map
*R*[*F*^2^ > 2σ(*F*^2^)] = 0.115	Hydrogen site location: inferred from neighbouring sites
*wR*(*F*^2^) = 0.346	H-atom parameters constrained
*S* = 1.12	*w* = 1/[σ^2^(*F*_o_^2^) + (0.1523*P*)^2^ + 2.4348*P*] where *P* = (*F*_o_^2^ + 2*F*_c_^2^)/3
1925 reflections	(Δ/σ)_max_ = 0.001
125 parameters	Δρ_max_ = 0.57 e Å^−^^3^
0 restraints	Δρ_min_ = −0.50 e Å^−^^3^

### Fractional atomic coordinates and isotropic or equivalent isotropic displacement parameters (Å^2^)

**Table:**

	*x*	*y*	*z*	*U*_iso_*/*U*_eq_	
O1	−0.1219 (4)	0.1464 (4)	0.8109 (6)	0.0463 (11)	
H1O	−0.1354	0.0584	0.8303	0.069*	
O2	0.5690 (4)	0.3709 (3)	0.4582 (5)	0.0401 (10)	
N1	0.0051 (5)	0.1629 (3)	0.7433 (6)	0.0307 (10)	
N2	0.1958 (4)	0.4074 (3)	0.6374 (5)	0.0293 (9)	
N3	0.3189 (4)	0.4064 (3)	0.5780 (5)	0.0280 (9)	
H3N	0.3551	0.4681	0.5396	0.034*	
C1	0.0281 (5)	0.2679 (4)	0.7208 (6)	0.0272 (10)	
C2	−0.0687 (6)	0.3628 (4)	0.7597 (8)	0.0377 (12)	
H2A	−0.1725	0.3547	0.6903	0.056*	
H2B	−0.0268	0.4364	0.7360	0.056*	
H2C	−0.0700	0.3594	0.8781	0.056*	
C3	0.1637 (5)	0.2962 (4)	0.6571 (6)	0.0269 (10)	
C4	0.2704 (5)	0.2239 (4)	0.6090 (6)	0.0267 (10)	
C5	0.2749 (6)	0.0955 (4)	0.6118 (7)	0.0337 (11)	
H5A	0.1801	0.0661	0.6327	0.050*	
H5B	0.3610	0.0697	0.7009	0.050*	
H5C	0.2865	0.0671	0.5037	0.050*	
C6	0.3685 (5)	0.2991 (4)	0.5583 (6)	0.0273 (10)	
C7	0.5069 (6)	0.2844 (4)	0.4959 (7)	0.0320 (11)	
C8	0.5688 (6)	0.1672 (4)	0.4798 (7)	0.0367 (12)	
H8A	0.6503	0.1722	0.4210	0.055*	
H8B	0.4873	0.1181	0.4165	0.055*	
H8C	0.6091	0.1348	0.5915	0.055*	

### Atomic displacement parameters (Å^2^)

**Table:**

	*U*^11^	*U*^22^	*U*^33^	*U*^12^	*U*^13^	*U*^23^
O1	0.043 (2)	0.042 (2)	0.059 (3)	−0.0029 (16)	0.0225 (19)	0.0047 (19)
O2	0.0369 (19)	0.0322 (19)	0.055 (2)	−0.0066 (15)	0.0191 (17)	0.0020 (17)
N1	0.0314 (19)	0.0256 (19)	0.040 (2)	−0.0020 (15)	0.0179 (16)	−0.0007 (16)
N2	0.035 (2)	0.0204 (19)	0.035 (2)	0.0004 (15)	0.0147 (17)	0.0016 (15)
N3	0.0322 (19)	0.0196 (18)	0.034 (2)	−0.0034 (14)	0.0121 (16)	0.0008 (15)
C1	0.0224 (19)	0.027 (2)	0.032 (2)	0.0001 (16)	0.0058 (17)	0.0007 (18)
C2	0.037 (3)	0.025 (2)	0.055 (3)	0.0029 (19)	0.020 (2)	0.000 (2)
C3	0.032 (2)	0.021 (2)	0.029 (2)	0.0015 (16)	0.0111 (18)	0.0023 (17)
C4	0.028 (2)	0.022 (2)	0.032 (2)	0.0019 (16)	0.0101 (17)	0.0022 (18)
C5	0.042 (3)	0.019 (2)	0.045 (3)	0.0005 (18)	0.021 (2)	0.004 (2)
C6	0.029 (2)	0.021 (2)	0.032 (2)	0.0013 (16)	0.0086 (18)	0.0032 (18)
C7	0.034 (2)	0.027 (2)	0.038 (3)	−0.0040 (18)	0.016 (2)	0.000 (2)
C8	0.031 (2)	0.031 (3)	0.052 (3)	0.0032 (19)	0.018 (2)	0.002 (2)

### Geometric parameters (Å, °)

**Table:**

O1—N1	1.410 (5)	C2—H2C	0.9800
O1—H1O	1.0542	C3—C4	1.413 (6)
O2—C7	1.234 (6)	C4—C6	1.386 (6)
N1—C1	1.267 (6)	C4—C5	1.503 (6)
N2—N3	1.324 (5)	C5—H5A	0.9800
N2—C3	1.353 (6)	C5—H5B	0.9800
N3—C6	1.357 (6)	C5—H5C	0.9800
N3—H3N	0.8839	C6—C7	1.479 (6)
C1—C3	1.487 (6)	C7—C8	1.500 (7)
C1—C2	1.498 (6)	C8—H8A	0.9800
C2—H2A	0.9800	C8—H8B	0.9800
C2—H2B	0.9800	C8—H8C	0.9800
			
N1—O1—H1O	109.3	C3—C4—C5	127.7 (4)
C1—N1—O1	111.7 (4)	C4—C5—H5A	109.5
N3—N2—C3	105.2 (4)	C4—C5—H5B	109.5
N2—N3—C6	112.8 (4)	H5A—C5—H5B	109.5
N2—N3—H3N	123.1	C4—C5—H5C	109.5
C6—N3—H3N	123.4	H5A—C5—H5C	109.5
N1—C1—C3	116.5 (4)	H5B—C5—H5C	109.5
N1—C1—C2	124.1 (4)	N3—C6—C4	107.2 (4)
C3—C1—C2	119.3 (4)	N3—C6—C7	118.9 (4)
C1—C2—H2A	109.5	C4—C6—C7	133.9 (4)
C1—C2—H2B	109.5	O2—C7—C6	118.1 (4)
H2A—C2—H2B	109.5	O2—C7—C8	121.6 (4)
C1—C2—H2C	109.5	C6—C7—C8	120.3 (4)
H2A—C2—H2C	109.5	C7—C8—H8A	109.5
H2B—C2—H2C	109.5	C7—C8—H8B	109.5
N2—C3—C4	111.1 (4)	H8A—C8—H8B	109.5
N2—C3—C1	118.5 (4)	C7—C8—H8C	109.5
C4—C3—C1	130.4 (4)	H8A—C8—H8C	109.5
C6—C4—C3	103.8 (4)	H8B—C8—H8C	109.5
C6—C4—C5	128.5 (4)		
			
C3—N2—N3—C6	−0.1 (5)	C1—C3—C4—C5	−0.7 (9)
O1—N1—C1—C3	177.3 (4)	N2—N3—C6—C4	−0.1 (6)
O1—N1—C1—C2	−0.6 (7)	N2—N3—C6—C7	−178.9 (4)
N3—N2—C3—C4	0.2 (5)	C3—C4—C6—N3	0.2 (5)
N3—N2—C3—C1	−179.2 (4)	C5—C4—C6—N3	179.9 (5)
N1—C1—C3—N2	−177.5 (4)	C3—C4—C6—C7	178.7 (5)
C2—C1—C3—N2	0.5 (7)	C5—C4—C6—C7	−1.6 (9)
N1—C1—C3—C4	3.2 (8)	N3—C6—C7—O2	−2.6 (8)
C2—C1—C3—C4	−178.8 (5)	C4—C6—C7—O2	179.0 (5)
N2—C3—C4—C6	−0.3 (5)	N3—C6—C7—C8	177.3 (5)
C1—C3—C4—C6	179.0 (5)	C4—C6—C7—C8	−1.0 (9)
N2—C3—C4—C5	−180.0 (5)		

### Hydrogen-bond geometry (Å, °)

**Table:**

*D*—H···*A*	*D*—H	H···*A*	*D*···*A*	*D*—H···*A*
O1—H1O···N2^i^	1.05	1.89	2.932 (6)	170
N3—H3N···O2^ii^	0.88	2.00	2.840 (5)	157

Symmetry codes: (i) −*x*, *y*−1/2, −*z*+3/2; (ii) −*x*+1, −*y*+1, −*z*+1.
